# Occurrence of *Fasciola* (Digenea: Fasciolidae) Species in Livestock, Wildlife and Humans, and the Geographical Distribution of Their Intermediate Hosts in South Africa—A Scoping Review

**DOI:** 10.3389/fvets.2022.935428

**Published:** 2022-07-20

**Authors:** Ignore Nyagura, Mokgadi Pulane Malatji, Samson Mukaratirwa

**Affiliations:** ^1^School of Life Sciences, College of Agriculture, Engineering and Science, University of KwaZulu-Natal, Durban, South Africa; ^2^Foundational Research and Services, South African National Biodiversity Institute, Pretoria, South Africa; ^3^One Health Centre for Zoonoses and Tropical Veterinary Medicine, Ross University School of Veterinary Medicine, Basseterre, Saint Kitts and Nevis

**Keywords:** fasciolosis, wildlife, human, livestock, intermediate hosts, distribution, prevalence, South Africa

## Abstract

This review was conducted to provide an update on the status of the occurrence of *Fasciola* species in livestock, wildlife and humans, and the geographical distribution of snail intermediate host (IH) species in South Africa. The literature search was conducted on four electronic databases using the Boolean operators in combination with predetermined search terms for thematic analysis. Results showed that *Fasciola* species have been reported in six out of nine provinces of South Africa in the last six decades (1960–2021), with both *F. hepatica* and *F. gigantica* infecting vertebrate hosts and *F. hepatica* and *Fasciola* spp infecting humans. Results also showed that most studies relied on morphological identification of eggs and flukes without molecular confirmation, which might have led to the misidentification of specimens, especially when immature. *Fasciola hepatica* has been documented in Limpopo, Mpumalanga, and KwaZulu-Natal provinces. The occurrences of *Galba truncatula* as the probable snail IH for *F. hepatica* in the three provinces has been documented while *Pseudosuccinea columella* has only been documented in Mpumalanga and KwaZulu-Natal provinces. The occurrence of *F. gigantica* to date has been reported in Mpumalanga and KwaZulu-Natal provinces, with overlapping distribution with *F. hepatica*. *Radix natalensis*, the main IH of *F. gigantica* has been documented in all the three provinces, while the two alien *Radix* species (*R. auricularia* and *R. rubiginosa*) were documented in KwaZulu-Natal province and have been implicated elsewhere with the transmission of *F. gigantica*. The presence of *Fasciola* spp eggs and antibodies in humans were documented in the Eastern Cape and the Western Cape provinces, where both *P. columella* and *G. truncatula* are known to be present. The prevalence of Fasciola spp infection in livestock ranged from 9.1 to 37.67 %, with an estimated annual financial loss ranging from R44930.26-129901 in cattle production in the Eastern Cape province of South Africa. This review reaffirms the scarcity of information on the occurrence and burden of fasciolosis in South Africa, and further highlights the importance of future research covering all provinces of the country and assessing the public health significance of the disease in resource-poor livestock communities in the areas where the parasite is endemic.

## Introduction

Fasciolosis is an important food- and water-borne parasitic zoonosis caused by the liver flukes of the genus *Fasciola* (1–3). The two main species are *Fasciola hepatica* Linnaeus (1758) and *F. gigantica* Cobbold (1856) (1, 2, 4, 5), and the disease has been reported to affect a wide range of domestic and wildlife mammals, and humans as definitive hosts and infection rates in livestock in some endemic areas can reach up to 90% (6). According to Mas-Coma et al. (1), the global economic implications associated with the disease have been estimated to be US$3.2 billion annually. These production losses are due to reduced productivity, liver condemnation, and reduced carcass value, and mortality associated with fasciolosis (7, 8).

Fasciolosis has raised public health concerns due to its potential zoonotic nature (9). According to Mas-Coma et al. (6), human fasciolosis is emerging/re-emerging in many parts of the world, and has been reported to be endemic in many countries of the Middle East and North Africa (Ethiopia) (10) and South America (11). Nonetheless, previous research indicated that an estimated 17 million individuals are infected, with 180 million persons at risk of infection globally (8, 12, 13).

The epidemiology of fasciolosis is associated with the ecological characteristics of the snail intermediate hosts implicated in the transmission of *Fasciola* species (14, 15). Lymnaeidae snail species act as intermediate hosts of *F. hepatica* and *F. gigantica*, and play an important role in the geographical distribution of these two species (1, 16–19). However, the susceptibility of these snail vectors to *Fasciola* species infections may vary depending on the immunological responses inherent to these snail species (3, 18) such as those related to the role played by IL-1 of *Biomphalaria glabrata* in resistance to *Schistosoma mansoni* infections (20). Over 20 Lymnaeidae species have been linked with the transmission of *Fasciola* species globally (8, 19), however, their capacity to sustain the developmental stages of the trematodes varies and their dispersion is determined by the climatic and ecological factors such as temperature, rainfall, habitat and soil stratum type (8). Owing to the wide range and distribution of *F. hepatica's* intermediate hosts, the parasite is widely distributed and commonly found in temperate areas (18). In African countries, this species has been found predominantly in cold (temperate) regions with high altitudes in Ethiopia (1,800 m above the sea level) (21, 22), Tanzania (3,000 m above the sea level) (23), and Uganda (3,500 m above the sea level) (24). According to Malatji et al. (4), these high altitudes seem not to be conducive to the survival of *Radix natalensis* which is the main intermediate host of *F. gigantica*. *Fasciola gigantica* has been reported in areas of lower altitude of around 1,200 m above the sea level in Ethiopia, and between 1,000 and 1,500 m above sea level in Uganda (4, 25).

Previous studies have revealed that both *F. hepatica* and *F. gigantica* occur in South Africa (4), and infections in definitive hosts and intermediate hosts have been reported in six of nine provinces of South Africa (4, 26–28). Reports have also shown that these two species have a geographical overlap in their distribution in Mpumalanga and KwaZulu-Natal provinces (4, 27–29). *Fasciola gigantica* was reported to infect cattle (4, 27–29), while *F. hepatica* infections were documented in wildlife (4, 30), cattle (4, 27–29, 31), and horses (32). There have also been cases of human infections in the Western Cape (33) and Gauteng (34) provinces. According to de Kock and Wolmarans (35), the low number of reported human cases in South Africa as compared to the other countries such as Egypt and Ethiopia is likely due to underreporting.

Nonetheless, the availability of information on the prevalence of fascioliasis in livestock (31, 32, 36) and wildlife (30) in South Africa is still limited and fragmented and more so, there is a need to quantify economic losses and the public health risk due to fasciolosis in South Africa. Therefore, the aim of this study was to review the information available to date on the prevalence and burden of *Fasciola* spp infection in livestock, wildlife and human, and the geographical distribution of snail IH species in South Africa. Furthermore, the information generated from the study will create an awareness among policy makers and various stakeholders, of the importance of the disease in livestock and wildlife and its public health importance in South Africa.

## Materials and Methods

### Scoping Review

Peer-reviewed research articles explicitly reporting on the occurrence, infection, and economic burden of *Fasciola* spp in livestock, wildlife, and humans, and the geographical distribution of their snail intermediate host species in South Africa, were reviewed following the scoping review framework as outlined by Arksey and O' Malley (37). The process included the following stages: (i) identifying the research questions; (ii) identifying relevant literature; (iii) literature selection; (iv) charting the data; and (v) collating, summarizing and reporting the results.

The following questions were identified: (i) What is the occurrence and geographical distribution of *Fasciola* spp in South Africa? (ii) What is the prevalence of *Fasciola* spp in livestock, wildlife, and humans in South Africa? (iii) What is the economic burden of fasciolosis in livestock and wildlife in South Africa? (iv) What is the occurrence and geographical distribution of intermediate hosts of *Fasciola* spp in South Africa?

### Search Strategy

Literature search was conducted on Google Scholar, PubMed, and SCOPUS using the Boolean operators (OR, AND) and the following terms: *Fasciola* OR fasciolosis AND livestock OR wildlife OR human, fasciolosis AND Prevalence, fasciolosis AND economic loss, *Fasciola* AND *Lymnaea* OR *Radix* OR *Galba* OR *Pseudosuccinea* species in South Africa. Literature search was limited to studies published from 1960 to 2021. Studies were initially identified by screening their titles and abstracts. Furthermore, the reference lists of the selected articles were screened as potential leads for additional relevant studies to include in the review. Full-text articles were retrieved and managed on Endnote reference manager version x9 (Clarivate Analytics, Philadelphia, PA, USA).

### Selection Criteria

The review specifically included articles published in peer-reviewed journals reporting on any of the following: (i) the occurrence and prevalence of fasciolosis in humans, livestock, and wildlife; (ii) the economic burden associated with fasciolosis in livestock and wildlife; (iii) and the geographical distribution of *Fasciola* spp intermediate host snail species. (iv) All the studies should have been conducted in South Africa and published in English.

Studies were excluded if they, (i) reported infections in the intermediate hosts by other trematodes other than *Fasciola* spp; (ii) were books, review papers and dissertations/thesis; (iii) they did not contribute toward answering the review questions; and (iv) were conducted outside South Africa.

### Charting, Collating, and Summarizing the Data

Data were extracted from the articles that met the inclusion criteria. Information of the author(s), year of publication, aims or objectives of the study, study location, the outcomes of the study, and any information that was relevant to the main objective of the review were recorded on a spreadsheet.

## Results

**Figure 1:**
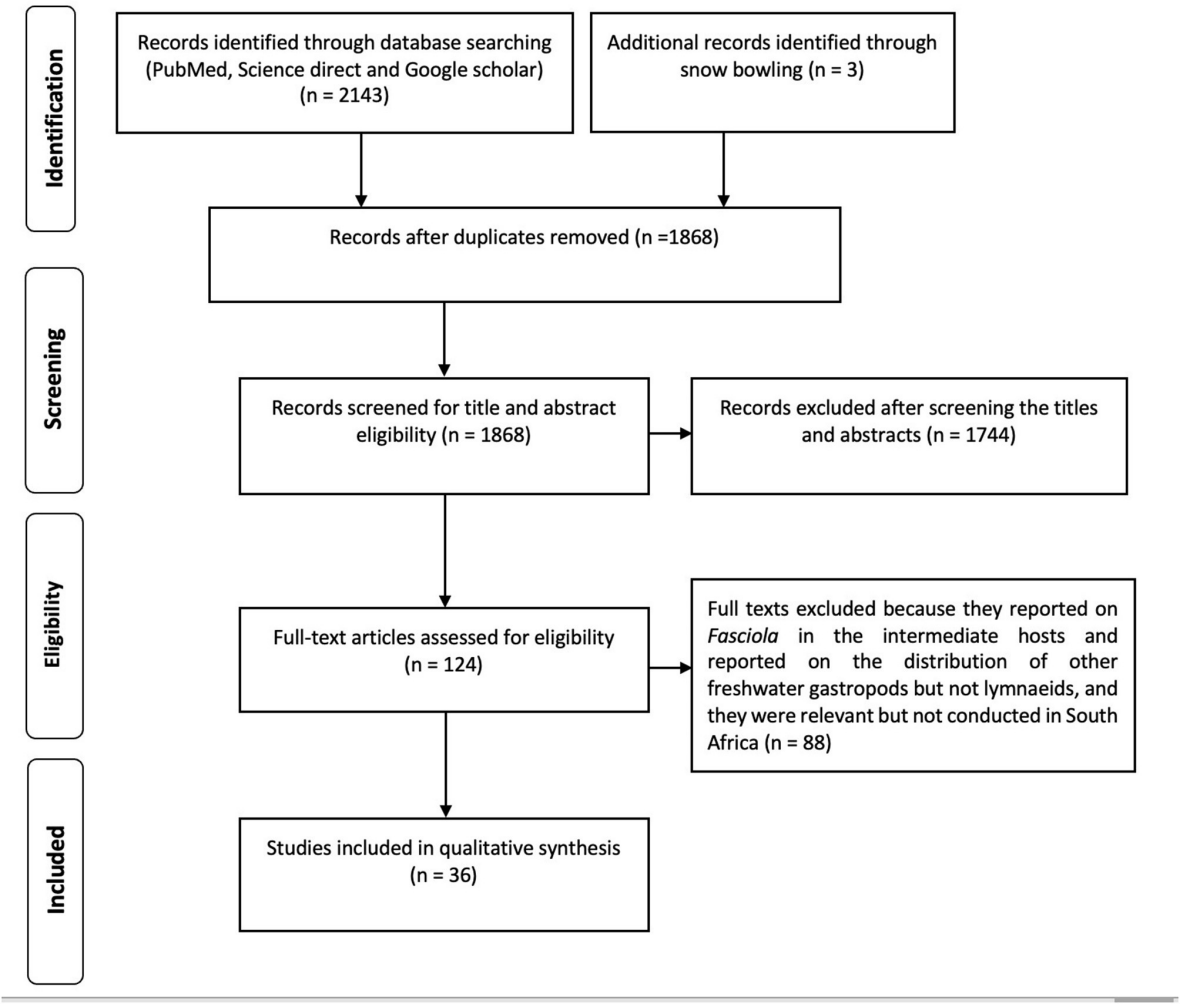
Search flow and selection processes.

A total of 2,143 records were identified through database searching (PubMed, Science direct, and Google Scholar), and three additional articles were identified through screening the bibliography of included articles (Figure 1). Of these, 278 articles were excluded due to duplications, and the title and abstracts of the remaining 1,868 articles were screened and 1,744 were deemed ineligible and excluded. Full-text reprints of 124 articles assessed for eligibility, and 88 articles were excluded because they did not explicitly report on the distribution of *Fasciola* spp and their intermediate hosts (*Radix* spp, *Galba* sp, *Pseudosuccinea* sp or *Lymnaea* spp), prevalence of fasciolosis in humans, livestock and wildlife, the economic loss associated with fasciolosis in livestock and wildlife in South Africa, or did not contribute toward answering the review questions. The remaining 36 articles met the inclusion criteria in this review.

Of the 36 studies, 11 studies reported on *Fasciola*, while 25 reported on the occurrence of the intermediate snail hosts (Tables 1, 2, respectively). From the 11 studies reporting on *Fasciola* pp, three (*n* = 3) studies reported on *F. hepatica* alone, while five (*n* = 5) could not identify up to species level, and three (*n* = 3) documented on both *F. hepatica* and *F. gigantica* (Table 1). Fasciolosis was documented more in cattle (bovine) (*n* = 7), followed by human (*n* = 2), while infections in the horse (*Equus caballus*) and kudu (*Tragelaphus strepsiceros*) were documented in one study each. Only four (*n* = 4) studies reported on the infection burden (prevalence) of fasciolosis (Table 3), while two studies (*n* = 2) highlighted the financial loss associated with fasciolosis in cattle (Table 4).

**Table 1:** Summary of studies which reported *Fasciola* spp. infection in livestock, wildlife and humans in different provinces of South Africa (1960–2021).

**Reference**	**Aim/objectives**	**Province**	**Host**	**Diagnostic method**	***Fasciola* species**	**Outcomes**
Haridwal et al. (28)	To identify *Fasciola* species collected from cattle slaughtered at abattoirs located in Mpumalanga and KwaZulu-Natal provinces of South Africa where *F. hepatica* and *F. gigantica* are co-endemic.	KwaZulu-Natal, Mpumalanga	Cattle	Fluke morphology and molecular	*F. gigantica, F. hepatica*	*- F. gigantica* and *F. hepatica* were co-endemic in Mpumalanga province, while only *F. hepatica* was found in KwaZulu-Natal province. - 15.5% (11/71) *F. hepatica* and 8.6% (6/71) *Fasciola* sp. were found with little to no sperms.
Chikowore et al. (27)	To differentiate and describe the phylogenetic background of *Fasciola* spp. isolated from cattle slaughtered at three abattoirs in the Mpumalanga and KwaZulu-Natal provinces of South Africa.	KwaZulu-Natal, Mpumalanga	Cattle	Molecular	*F. gigantic*a, *F. hepatica*	- Both *Fasciola* spp. were documented. *- Fasciola hepatica* was shown to be more prevalent than *F. gigantica*
Jaja et al. (36)	To determine the seasonal prevalence and risk factors associated with *Fasciola* infection in cattle.	Eastern Cape	Cattle	Coprology, antemortem and post-mortem survey	*Fasciola* spp	- The infection with *Fasciola* spp was higher in the summer than in the winter - There was a positive association between the prevalence of fasciolosis and poor body condition in study animals.
Jaja et al. (38)	To assess the prevalence and monetary losses associated with *Fasciola* infection at three abattoirs in Eastern Cape Province.	Eastern Cape	Cattle	Retrospective financial data	*Fasciola* spp	- Total financial loss due to *Fasciola* infection from three abattoirs was ZAR44, 930 (USD3 456.20) - The prevalence of *Fasciola* was higher and more livers were condemned during the wet and warm season.
Jaja et al. (39)	To evaluate causes of liver condemnation and the financial loss of condemnation	Eastern Cape	Cattle	Retrospective study (RS) and post-mortem meat inspection	*Fasciola* spp	- A retrospective study (RS) revealed that 5.95% (*n* = 10,276), 4.48% (*n* = 14,625), and 2.7% (n = 26,401) of livers were condemned due to fasciolosis for year 2010, 2011, and 2012, respectively.
Mucheka et al. (29)	To identify and determine the genetic diversity of *Fasciola* species in cattle from Zimbabwe, the KwaZulu-Natal and Mpumalanga provinces in South Africa and selected wildlife hosts from Zimbabwe	KwaZulu-Natal, Mpumalanga	Cattle	Molecular	*F. gigantica, F. hepatica*	- Validated the occurrence of both *Fasciola* species using molecular using molecular. *- Fasciola hepatica* was shown to be more prevalent than *F. gigantica*
Black et al. (33)	To report cases of human fascioliasis in South Africa	Western Cape	Human	IFAT	*Fasciola* sp	- Human infection with *Fasciola* spp. were recorded in two human cases.
Van Wyk and Boomker (30)	To determine the types and numbers of helminth that occur in different wildlife hosts in Limpopo as well as whether any zoonotic helminths were present.	Limpopo	Kudu (*Tragelaphus strepsiceros)*	Antemortem and post-mortem	*F. hepatica*	- Only one Kudu was found infected with *Fasciola hepatica* during antemortem and post-mortem assessment.
Ndlovu et al. (31)	To determine monthly changes in body condition scores, body weights and on the prevalence of internal parasites in Nguni, Bonsmara and Angus steers raised on sweetveld	Eastern Cape	Cattle	Coprology	*Fasciola* spp	- Nguni steers had the lowest parasite infestation levels, with the Bonsmara being more susceptible than the other two breeds
Alves et al. (32)	To assess the susceptibility of horses to artificial infestations with both *Fasciola* spp.	Mpumalanga	Horse	Coprology	*F. hepatica*	- Horses had a high level of resistance to South African *Fasciola* spp.
Scott and Irving (34)	To determining the cause of illness	Gauteng	Human	Coprology	*F. hepatica*	*- Fasciola hepatica* infection was reported and the identity is with caution as no details were provided on how the parasite was identified.

**Table 2:** Information from reviewed publications on geographical distribution of snail intermediate hosts of *Fasciola* spp. in South Africa.

**Species**		**Status**	**Province**	**Habitat type**	**Reference**
*Galba truncatula*		Native	Limpopo, Gauteng, Mpumalanga, North West, Free State, KwaZulu-Natal, Eastern Cape, Western Cape	Channel, concrete dam, dam, ditch, irrigation furrow, pond river, spring, stream, swamp, waterhole	(35, 40, 41)
*Radix natalensis*		Native	Limpopo, Gauteng, Mpumalanga, North West, Free State, Northern Cape, KwaZulu-Natal, Eastern Cape, Western Cape	Channel, concrete dam, dam, ditch, fountain, lakes, farm, pan, pool, river, spruit, stream, waterhole	(8, 41–54)
*Pseudosuccinea columella*		Alien invasive	Limpopo, Gauteng, Mpumalanga, North West, Free State, KwaZulu-Natal, Eastern Cape, Western Cape	Channel, concrete dam, dam, ditch, fountain, irrigation furrow, lakes, marsh/ swamp, pan, pit, pool, pond, river, spruit, spring, waterhole, wetlands	(8, 26, 35, 41, 44–46, 52, 54–60)
*Radix auricularia*		Alien	KwaZulu-Natal	River, dam, pond	(8)
*Radix rubiginosa*		Alien	KwaZulu-Natal	Tanks, drains, reservoirs, channels and shallow trays storing plants	(61)

**Table 3:** Information from reviewed publications on the prevalence of *Fasciola* spp. in humans, wildlife and livestock from South Africa.

**Province (s)**	**Host**	**No. examined**	**No. positive**	**Prevalence (%)**	**Species of infection**	**Diagnostic method**	**Reference**
Eastern Cape	Cattle	1,120	420	37.5	*Fasciola* spp.	Morphology (post-mortem)	(36)
Eastern Cape	Cattle	1,120	270	24.11	*Fasciola* spp.	Coprology	(36)
Limpopo	Kudu	8	1	12.5	*F. hepatica*	Morphology (post-mortem)	(30)
Eastern Cape	Cattle	55	9	16.33	*Fasciola* spp.	Coprology	(31)
Mpumalanga	Horse	11	1	9.1	*F. hepatica*	Coprology	(32)

**Table 4:** Information from reviewed publications on the economic burden due to liver condemnation caused by fasciolosis in cattle reported in South Africa.

**Reference**	**Year**	**Province**	**Host**	**No. of livers examined**	**No. of livers condemned**	**Prevalence (%)**	**Annual loss**		
							**Partial liver condemnation**	**Whole liver condemnation**	**Total loss**
Jaja et al. (38)	2010–2012	Eastern Cape	Cattle	78,728	2,249	0.03	–	–	ZAR129 901.00
Jaja et al. (38)	2013–2014	Eastern Cape	Cattle	3,142	716	0.228	ZAR19 700.00	–	ZAR44 930.26
Jaja et al. (38)	2013–2014	Eastern Cape	Cattle	3,142	459	0.146	–	ZAR25 230.26	ZAR44 930.26
Jaja et al. (39)	2013	Eastern Cape	Cattle	1,374	156	11.35	–	–	ZAR10 526.40

### Geographical Distribution of *Fasciola* spp and Lymnaeids in South Africa

**Figure 2:**
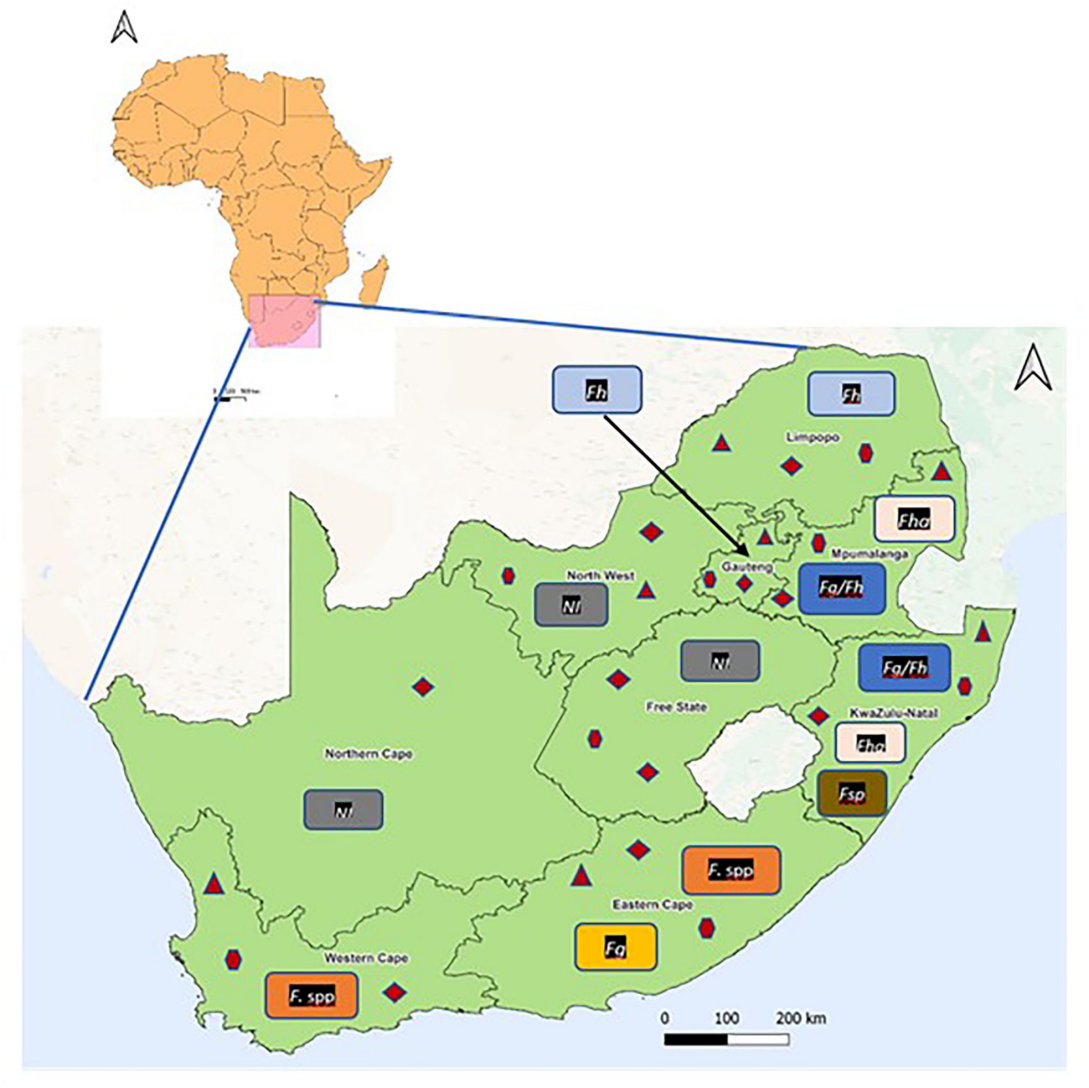
Reported geographical occurrence of *Fasciola* species and their lymnaeid intermediate hosts in the provinces of South Africa. 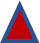 = *Galba truncatula*; 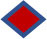 = *Radix natalensis*; 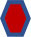 = *Pseudosuccinea columella*. Fg/Fh, *Fasciola gigantica* and *F. hepatica*; Fg, *F. gigantica*; Fh, *F. hepatica*; Fha, *Fasciola hepatica* aspermic; *Fasciola* spp, *Fasciola* species; *Fasciola* sp, Suspected *Fasciola* hybrid; NI, No information available on *Fasciola* species.

Results showed that fasciolosis has been reported in cattle, horses, kudu, and humans in Limpopo, Gauteng, Mpumalanga, KwaZulu-Natal, Eastern Cape, and Western Cape provinces (Table 1; Figure 2). *Fasciola hepatica* infection was documented in Kudu in Limpopo based on the morphological identification of the adult flukes post-mortem, in horses and humans from Mpumalanga and Gauteng provinces, respectively, based on the coprological technique, and cattle in Mpumalanga and KwaZulu-Natal provinces based on the morphological and molecular techniques. Results also showed the presence of the aspermic populations of *F. hepatica* in Mpumalanga and KwaZulu-Natal provinces, documented in the cattle and identified based on the morphological and molecular techniques (28). Infections due to *F. gigantica* were identified and recorded in cattle in Mpumalanga and KwaZulu-Natal, in conjunction with *F. hepatica* based on the morphological and molecular techniques (Table 1). *Fasciola* spp infections were reported in cattle in the Eastern Cape province based on coprological, antemortem, and post-mortem assessment, and in humans in the Western Cape province based on the antibody test (IFAT).

In total, five lymnaeid snail species, namely, *Radix natalensis* (Krauss, 1848), *Galba truncatula* (Muller, 1774), *Pseudosuccinea columella* (Say, 1817), *Radix auricularia* (Linnaeus, 1758), and *Radix rubiginosa* (Michelin, 1831) have been reported in South Africa (Table 2; Figure 2). *Radix natalensis* was documented in all the nine provinces of South Africa, *viz* Limpopo, Gauteng, Mpumalanga, North West, Free State, Northern Cape, KwaZulu-Natal, Eastern Cape, and Western Cape provinces. *Galba truncatula* was reported in Limpopo, Gauteng, Mpumalanga, North West, Free State, KwaZulu-Natal, Eastern Cape, and Western Cape provinces (Figure 2). Reviewed publications show that *P. columella* was distributed in the Limpopo, Mpumalanga, KwaZulu-Natal, Eastern Cape, and Western Cape provinces. In the Eastern Cape and KwaZulu-Natal provinces, this species was found naturally infected with *F. gigantica* and *Fasciola* sp (26). *Radix rubiginosa* and *Radix auricularia* were reported for the first time in South Africa in KwaZulu-Natal province.

### Prevalence of *Fasciola* spp in Cattle

From the studies reviewed, the prevalence of *Fasciola hepatica* infection ranged from 9.1% in horses in Mpumalanga based on coprology, to 12.5% in Kudu from Limpopo province, based on antemortem and post-mortem assessment (Table 3). The prevalence of *Fasciola* spp infection in cattle ranged from 16.33 to 37.67% based on coprology (31), and post-mortem survey (36) in the Eastern Cape province.

Host-based risk factors associated with the prevalence of fasciolosis in South Africa were only assessed in one study from the Eastern Cape province (36). The results showed that for both low-throughput abattoir (LTPA) and high-throughput abattoirs (HTPA), the prevalence of fasciolosis infection was generally higher in young animals (HTPA1 = 30.80%, HTPA2 = 23.20%, and LTPA = 18.20%) as compared with the older animals (HTPA1 = 8.80%, HTPA2 = 14.50%, LTPA = 14.10%). The HTPA1 showed no differences in the prevalence of infection between sex. However, HTPA2 showed high-infection rate in females (26.0%) than males (11.20%), and the LTPA showed a high prevalence in males (29.10%) as compared with females (3.20%). The HTPA abattoirs recorded high prevalence in animals with low body condition score (BCS) (26.60; 18.50%) followed by moderate BCS (10.40; 13.0%), whereas the prevalence of infections in the LTPA was high in animals with moderate BCS (15.90%) followed by those with good BCS (9.10%) (36).

### Economic Losses Due to *Fasciola* spp Infection in Cattle

Results showed that the financial impact due to fasciolosis from the cattle abattoir study was due to the partial or whole liver condemnation. Financial loss due to partial liver condemnation was estimated at ZAR19 700.00 from 716 condemned livers from 3,142 cattle slaughtered, and ZAR25 230.26 from full liver condemnation at cattle abattoirs in 2013 and 2014 in the Eastern Cape province (Table 4). The annual financial losses associated with the liver condemnation and carcass weight loss due to the chronic form of fasciolosis were estimated as ZAR129 901.00 from 2010 to 2012 and ZAR44 930.26 from 2013 to 2014 based on abattoir slaughter records (38). In addition, a financial survey from July to December showed that ZAR10 526.4 was lost due to liver condemnation caused by fasciolosis (39).

## Discussion

Results from reviewed studies showed that cases of *Fasciola* spp infections have been reported in Limpopo, Gauteng, Mpumalanga, KwaZulu-Natal, Eastern Cape and Western Cape provinces. Infections were reported in cattle, horse, kudu, and human, and corresponded with the distribution of the snail intermediate hosts implicated in the transmission of the species. *Fasciola hepatica* infections were documented in Limpopo, Gauteng, Mpumalanga, and KwaZulu-Natal provinces, where the native intermediate hosts of this species, *G. truncatula*, has been documented (40, 41). Furthermore, Haridwal et al. (28) also reported the presence of aspermic *Fasciola* sp specimens from *F. hepatica* population from cattle slaughtered at abattoirs in Mpumalanga and KwaZulu-Natal provinces. In addition, other snail species such as the native *R. natalensis* and the alien invasive *P. columella* were also documented in these provinces (32, 40, 41, 58), which explained the presence of *F. gigantica* in Mpumalanga and KwaZulu-Natal provinces and the overlapping distribution between the two species in these two provinces. Furthermore, *P. columella* has been previously found naturally infected with *F. gigantica* and *Fasciola* sp in KwaZulu-Natal (26). KwaZulu-Natal further documented two alien species, *R. auricularia* and *R. rubiginosa* which act as intermediate hosts of *F. gigantica* among other trematodes in their native origin and elsewhere (8, 61). Surprisingly, *F. gigantica* has not been documented in Limpopo and Gauteng, despite the presence of *R. natalensis* in these provinces.

Results also show that several studies did not identify the liver flukes up to species level. This was observed more especially in the Eastern Cape and Western Cape, where *Fasciola* spp infection was documented in cattle and humans, respectively. Lack of identification up to species level may have been attributed to the use of diagnostic tools such as coprology (31, 36, 38, 39) and antemortem and post-mortem assessment (36, 38, 39) in cattle and IFAT in humans (33) which can only confirm identity up to genus level. Considering that the intermediate hosts of both *F. hepatica* (*G. truncatula*) and *F. gigantica* (*R. natalensis*) including *P. columella* which have been confirmed to transmit *F. gigantica* in the Eastern Cape provinces, have been documented in these areas (26, 40, 59), it is possible that the *Fasciola* eggs or adult flukes reported could have been of any of the two *Fasciola* species.

Reviewed studies showed that although no peer-reviewed case reports or studies reported the occurrence of fasciolosis in some provinces, the intermediate hosts of these species were documented. This includes North West province, where the presence of *G. truncatula* and *P. columella* which are known intermediate host of *F. hepatica* and *R. natalensis* and *P. columella*, which are intermediate hosts of *F. gigantica* (41, 49, 56), and Free State province where *G. truncatula* (35, 40) and *P. columella* (55, 60) were previously documented. The presence of these snail species may indicate that fasciolosis in these provinces may be the presence, but unreported.

Only three cases of human fasciolosis have been documented in Gauteng (*n* = 1) and Western Cape (*n* = 2) provinces in South Africa from 1960 to 2013 (33, 34). Prior to 1960, only two cases in humans have been documented in the Eden district of the Western Cape province (62). According to Black et al. (33), the patient got infected by ingesting watercress purchased from the local markets and this corresponded with the observation made by Soliman (63) that infections in humans were linked to the consumption of watercress which is part of the regular diet of communities in several countries. Furthermore, more cases may be existing but a lack of awareness of the disease in the medical fraternity might be contributing to the low-human cases (33). This suggests that the Eden district of the Western Cape province may be a potential endemic area for human fasciolosis in South Africa.

According to Black et al. (33), the epidemiology of fasciolosis in livestock and humans in South Africa is still unknown. Results show that only four studies documented the prevalence of fasciolosis infection in horse, kudu, and cattle. Only one of eight Kudu (12.5%, 1/8) from Limpopo provinces was infected with *F. hepatica* (30). Although *F. hepatica* infections in Greater Kudu have only been reported in South Africa, *F. gigantica* infections have been documented in other southern African countries such as Zimbabwe (64) and Zambia (65). The low-infection rate may have been because fasciolosis is not common in kudus, due to browsing behavior which makes it less likely to get exposed to aquatic vegetations (66). Infection in these kudus may be attributed to the water source (dam), fed from a river, which was accessed by cattle and provided a favorable environment for freshwater snails that serve as IHs for the liver fluke (30).

Like kudu, only one (1/11, 9.1%) horse from Mpumalanga was found infected with *F. hepatica* (32). According to Alves et al. (32), the low-infection rate in horses may be due to the high-resistance level of horses to *Fasciola* spp strains present in South Africa after experimental infections failed to establish. Similar observations were made elsewhere, where horses were frequently resistant to infection with *Fasciola* (32, 67, 68), although high-infection rates have been documented especially in areas where the fasciolosis is endemic and horses share grazing with highly infected animals (69–71).

Prevalence of *Fasciola* spp infection in cattle ranged from 16.33 to 37.67%, although limited to the Eastern Cape province. The reviewed studies further indicated that the prevalence of fasciolosis is influenced by factors such as season, age, gender, and body condition scores of the animal. The prevalence was higher in the wet and warm season (31, 36), corresponding with the infection trend observed in Zimbabwe where the highest infection was in the month of February and December, and the lowest in September (72). Corresponding with the numerous studies from various countries, reviewed studies further showed that the infection rate was significantly higher in young animals than in old animals (73–75). However, only one study compared prevalence in males and female in cattle that showed high-prevalence of fasciolosis in males than females in the low-throughput abattoirs (LTPAs) which corresponded with reports from other studies (21, 74, 76–78), but the HTPA reported high infections in the females (36). Animals with poor body conditions scores showed to be more susceptible to infection (21, 74, 79, 80).

*Fasciola* infection causes enormous loss to the ruminant livestock production sector through liver condemnation, reduction in milk, meat and wool production, metabolic diseases, veterinary care, and mortality (81–84). Although several African countries have reported that millions of dollars were lost due to fasciolosis (72, 85, 86), the financial implication of fasciolosis in South Africa is still scanty (36). Results show that to date, only two studies assessed the economic loss associated with fasciolosis in South Africa with an annual financial loss associated with either partial or whole liver condemnation and carcass weight loss being reported (38, 39). However, these represent data from only three abattoirs in the Eastern Cape province of South Africa, and as such, cannot be extrapolated beyond this province.

## Conclusion

The review showed *Fasciola* spp have been documented in six provinces of South Africa in the last six decades, and their geographical distribution correspond with the presence of their intermediate hosts. The review also showed the presence of intermediate hosts of Fasciola spp in provinces where infections in vertebrate hosts have not been documented. Furthermore, fasciolosis in South Africa was documented in cattle, horse, kudu, and humans, and the number of studies is scanty considering the geographical size of South Africa, and the economic and veterinary public health importance of the disease. The review showed the scarcity of information or studies, especially, on the epidemiology and cost-benefit analysis in the control and prevention of the disease in the animal production sectors. Hence, the authors recommend more research on the epidemiology of the disease covering all provinces and designing effective prevention and control strategies for the disease targeting resource-poor livestock farmers whose livestock are mostly negatively affected by fasciolosis. Future studies could also include surveillance of human fasciolosis, more especially in endemic areas, including areas where cases have been documented previously.

## Author Contributions

SM and MM conceptualized the study. IN and MM developed the concept note. IN conducted the search, selected studies under MM's supervision, and wrote the first draft of the manuscript. All author's contributed to the article, agreed on the final draft and approved the submitted version.

## Funding

This project has received funding from the European Union's Horizon 2020 Research and Innovation Program under grant agreement No 101000365. The study also received financial assistance from SM's Research Productivity funds (UKZN).

## Conflict of Interest

The authors declare that the research was conducted in the absence of any commercial or financial relationships that could be construed as a potential conflict of interest.

## Publisher's Note

All claims expressed in this article are solely those of the authors and do not necessarily represent those of their affiliated organizations, or those of the publisher, the editors and the reviewers. Any product that may be evaluated in this article, or claim that may be made by its manufacturer, is not guaranteed or endorsed by the publisher.
